# Supplementary material for: The absence of SOX2 in the anterior foregut alters the esophagus into trachea and bronchi in both epithelial and mesenchymal components
Source: Biol Open. 2020 Feb 7;9(2):bio048728. doi: 10.1242/bio.048728 (PMC7044460; doi:10.1242/bio.048728)
Supplement: Supplementary information [file biolopen-9-048728-s1.pdf]

Biology Open (2020): doi:10.1242/bio.048728: Supplementary information

Figure S1

A

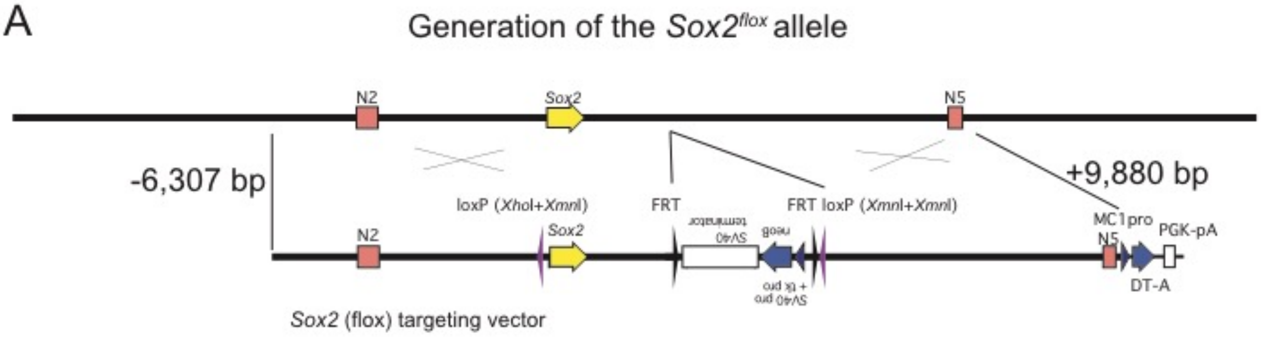

B

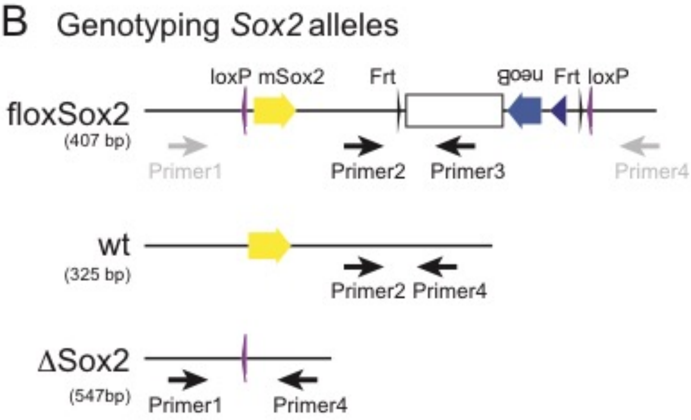

C Primer sequences

Primer 1: AGGAGTTGTCAAGGCAGAGAAGAGAGTG

Primer 2: GCATTGTGCGCTCTCATCACATTTGGAC

Primer 3: CTTGTGGTCAGTGTTCATCTGCTGACTG

Primer 4: TGGATAGAAGATGACCGAAACCAGGAGC

D

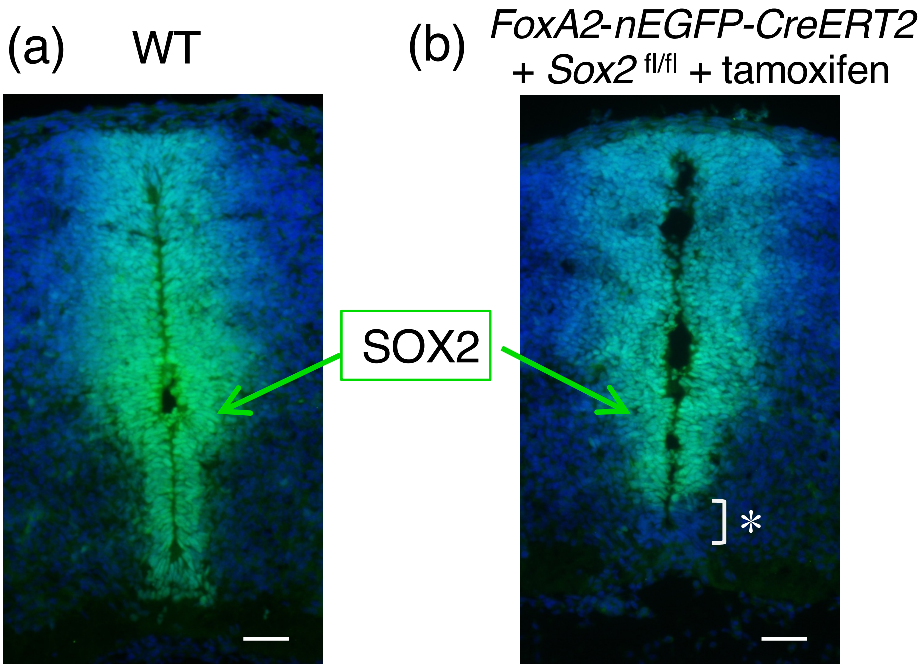

Biology Open • Supplementary information

Figure S1. Construction and genotyping of floxed *Sox2* alleles. **(A)** A scheme of generating the floxed *Sox2* allele. A targeting vector containing the STneoB cassette (Katoh et al., 1987) flanked by a pair of Flp recombinase target (FRT) was recombined with the mouse *Sox2* locus sequences in R1 ES cells (Nagy et al., 1993). **(B)** Mouse and embryo genotypes were determined using polymerase chain reaction (PCR) with primer pairs that gave PCR products of indicated sizes. Embryos carrying the homozygous floxed *Sox2* allele and the ROSA26 locus-inserted CreERT2 recombinase gene (Gt$(ROSA)26Sor^{tm1(cre/ERT2)Alj}$) (Cheng et al., 2010) showed the Δ*Sox2* genotype after the administration of tamoxifen to mothers on E7 and E8. **(C)** PCR primer sequences. **(D)** (a) Spinal cord of a WT embryo on E13 stained for SOX2 (green) and 4′,6-diamidino-2-phenylindole (blue). (b) Spinal cord of an embryo carrying homozygous floxed *Sox2* and *FoxA2-nEGFP-CreERT2* alleles treated with tamoxifen. Weak nEGFP fluorescence was quenched by treating the fixed specimen with ethanol. Note that SOX2 immunofluorescence was lost in the floor plate-proximal ventral portion of the spinal cord, as indicated by a bracket with an asterisk. Bars, 50 µm.

Biology Open • Supplementary information

Figure S2

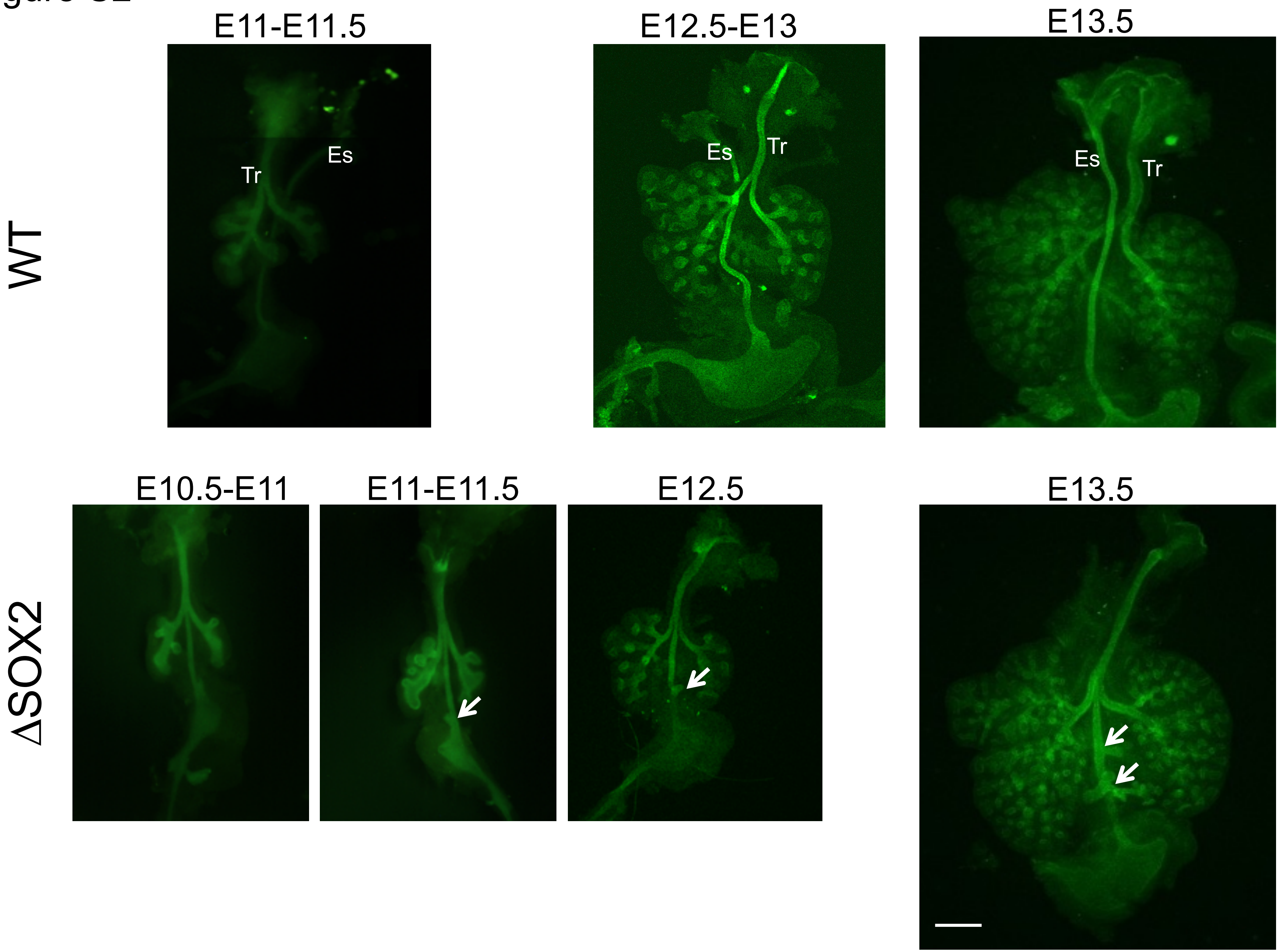

Biology Open • Supplementary information

Figure S2. Development of lung tissues from WT and ΔSOX2 AFG at developmental stages from E11 to E13.5. Lung tissue growth and branching patterns in ΔSOX2 were not affected. Occasionally, bronchiole tissues budded out of ΔSOX2 posterior AFG, as indicated by the arrows. Bar, 500 µm.

Figure S3

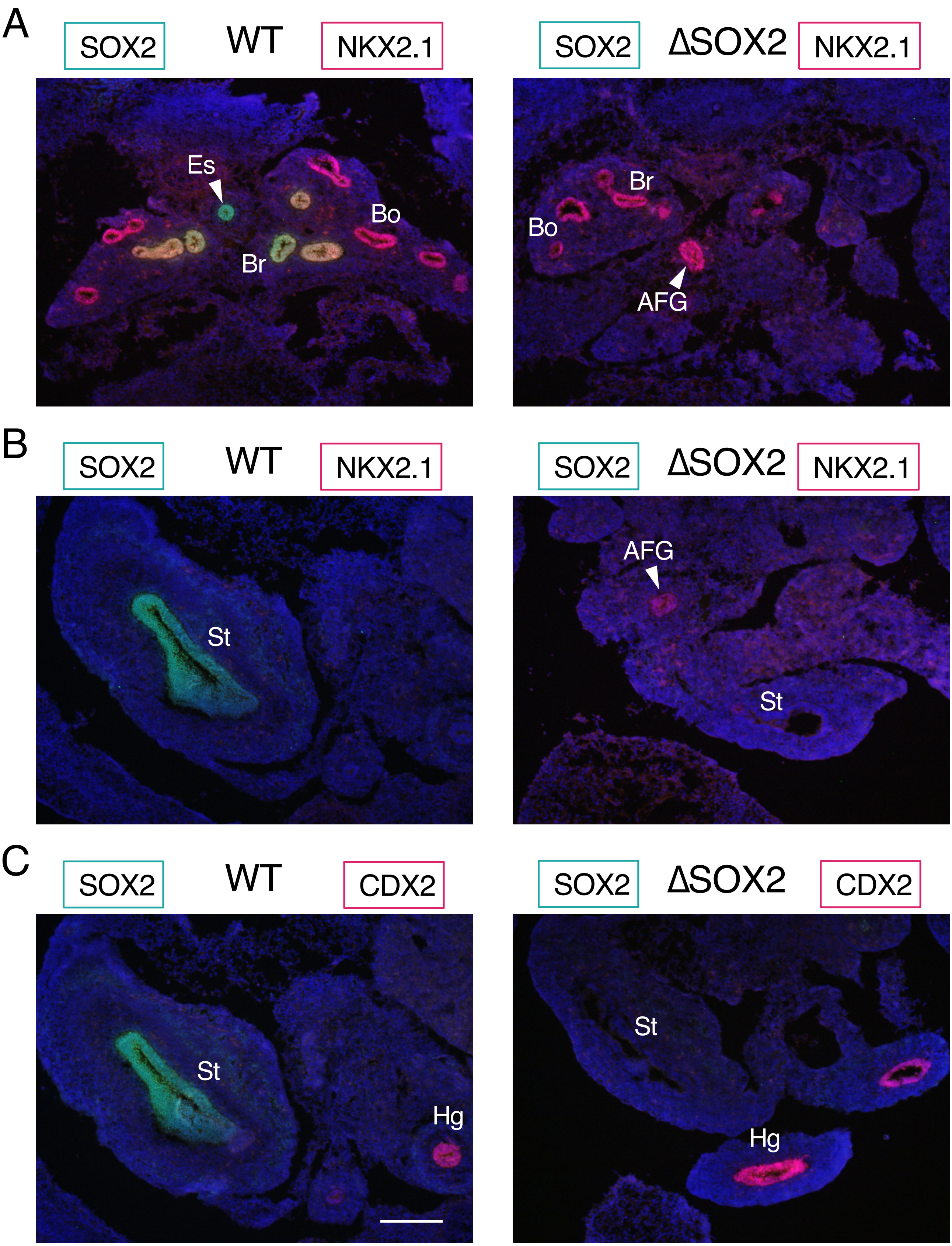

Biology Open • Supplementary information

Figure S3. Comparison of the expression of SOX2 (green) and NKX2.1/CDX2 (red) between WT and ΔSOX2 embryos at E13 in composite fluorescence images with DAPI (blue) (n = 3). Cryosections at lung **(A)** and stomach **(B and C)** axial levels were immunostained. Es, esophagus; Br, Bronchi; Bo, Bronchioles; AFG, AFG tube of SOX2-deficient embryo; St, stomach; Hg, hindgut. Bar, 200 μm. **(A)** WT esophagi expressed only SOX2, and bronchi expressed various levels of SOX2 and NKX2.1, whereas bronchioles expressed only NKX2.1. By contrast, in ΔSOX2 embryos, all endoderm-derived tubes at this level expressed NKX2.1. **(B and C)** Stomach epithelia expressed neither NKX2.1 nor CDX2 even in the absence of SOX2 expression.

Biology Open • Supplementary information

Table S1. Antibodies and immunostaining conditions.

Target protein	Primary antibodies	Supplier	Product number	Dilution
NKX2.1	Mouse monoclonal IgG	Thermo	MS-699-P0	200×
SOX2	Goat polyclonal IgG	R&D	AF2018	200×
Smooth muscle α-actin	Mouse monoclonal IgG	Sigma	A2547	2000×
SOX9	Rabbit polyclonal IgG	Millipore	AB5535	1000×
CDX2	Mouse monoclonal IgG	Abcam	ab157524	200×
GFP	Rabbit polyclonal IgG	MBL	598	100×
E-cadherin	Mouse monoclonal IgG	Abcam	ab76055	500×

Secondary antibodies were Alexa Fluor 568 Donkey Anti-mouse IgG (Abcam, ab175700), Alexa Fluor 488 Donkey Anti-rabbit IgG (Abcam, ab150061), or Alexa Flour 488 Donkey Anti-goat IgG (Abcam, ab150133) used at 200× dilution.

Table S2. Probes and *in situ* hybridization conditions.

Target transcript	RefSeq	Base positions	Length (bases)
Wnt4	NM_009523	46–1101	1056
Tbx4	NM_172798.1	1761–2576	816
Hoxb6	NM_008269.2	103–1146	1043

In situ hybridization of isolated internal organs was performed using digoxigenin-labeled probes and conditions described by Takemoto et al. (2011) with Proteinase K treatment at 5 μg/ml.
